# Modeling, Simulation, Experimentation, and Compensation of Temperature Effect in Impedance-Based SHM Systems Applied to Steel Pipes

**DOI:** 10.3390/s19122802

**Published:** 2019-06-22

**Authors:** Rothschild A. Antunes, Nicolás E. Cortez, Bárbara M. Gianesini, Jozue Vieira Filho

**Affiliations:** 1 Department of Information Technology, Federal Institute of Education, Science and Technology of Mato Grosso, Cuiabá, MT 78005-200, Brazil; 2 Department of Electrical Engineering, São Paulo State University (UNESP), Ilha Solteira, SP 15385-000, Brazil; jozue.vieira@unesp.br; 3 Department of Electrical Engineering, Federal University of Mato Grosso, Cuiabá, MT 78060-900, Brazil; nkortez@gmail.com (N.E.C.); bmgianesini@hotmail.com (B.M.G.); 4 Electrical Engineering Faculty, Federal University of Uberlândia, Uberlândia, MG 38400-902, Brazil; 5 Telecommunications and Aeronautical Engineering, São Paulo State University (UNESP), São João da Boa Vista, SP 13876-750, Brazil

**Keywords:** SHM, EMI, PZT, damage detection, finite elements, temperature compensation

## Abstract

Pipelines have been widely used for the transportation of chemical products, mainly those related to the petroleum industry. Damage in such pipelines can produce leakage with unpredictable consequences to the environment. There are different structural health monitoring (SHM) systems such as Lamb wave, comparative vacuum, acoustic emission, etc. for monitoring such structures. However, those based on piezoelectric sensors and electromechanical impedance technique (EMI) measurements are simple and efficient, and have been applied in a wide range of structures, including pipes. A disadvantage of such technique is that temperature changes can lead to false diagnoses. To overcome this disadvantage, temperature variation compensation techniques are normally incorporated. Therefore, this work has developed a complete study applied to damage detection in pipelines, including an innovative technique for compensating the temperature effect in EMI-based SHM and the modeling of piezoceramics bonded to pipeline structures using finite elements. Experimental results were used to validate the model. Moreover, the compensation method was tested in two steel pipes—healthy and damaged—compensating the temperature effect ranging from −40 °C to +80 °C, with analysis on the frequency range from 5 kHz to 120 kHz. The simulated and experimental results showed that the studies effectively contribute to the SHM area, mainly to EMI-based techniques.

## 1. Introduction

There are at least two main concerns when dealing with structures presently. First, the global and fast-paced growth of modern constructions, to cater to the most diverse needs of the population, demands an increasingly rigorous performance from engineering areas. Second, due to the law of supply and demand, the concern to reduce costs and deadlines for concluding those works may decrease rigor in executing projects and the lifespan of construction. Consequently, corrosion and material fatigue may accelerate the occurrence of structural damage [1]. Moreover, regarding modes of transportation, there is no doubt that pipelines are the best options to distribute water, gas, oil, and fluids in general, not only because they present lower costs when compared to terrestrial, railway, and air transport, but also because they are considered safe.

In this sense, structural health monitoring (SHM) is an area that has been growing significantly in the last few years since industrial engineers and academic researchers are becoming increasingly concerned with the health of structures such as pipelines. Therefore, they continuously seek systems and mechanisms to monitor structural health for detecting damage and/or gas or liquid leakage, to guarantee higher safety levels to users and the environment, as well as to reduce costs [2,3,4,5]. There are many methods according to the stages of SHM systems, but in the present work, the focus is the damage detection by means of the electromechanical impedance technique (EMI), which is based on low cost PZT (Pblead Zirconate Titanate) piezoelectric transducers. Such transducers are extremely sensitive in detecting local damage [2,4,6,7] and allow the implementation of non-invasive SHM systems. The basic principle of the EMI technique is to monitor the changes in the mechanical impedance of a structure, caused by alterations in its dynamic response, due to the presence of damage or even due to temperature variation. Considering that measuring the mechanical impedance of a structure is a complex task, PZT piezoelectric transducers are attached to the structure to monitor its mechanical impedance through measuring the electrical impedance, which is simpler to implement [8,9].

There are many studies and techniques to monitor the structural behavior of ducts and pipes based on EMI, in which the goal is to identify damage and/or leakage. One of the first studies involving pipelines was to assess a pipeline structure connected through flanges. The authors used a HP4194 impedance analyzer to measure the real part of the impedance, on a frequency range from 80 to 100 kHz, seeking to detect and analyze damage, as the screws of those flanges were removed [10]. In another work, tests were performed in the same pipeline structure, but this time using HP36665A impedance analyzer to detect five types of damage in the 35–47.8 kHz frequency range [11]. Both works presented good results, but did not explore the temperature variation effect. Another important work was developed using five MFC (Microfiber Composer) transducers, which are flexible and have similar characteristics to traditional piezoelectric ceramics. A tubular structure was monitored to analyze pipe-coupling flanges where the screws were loose to generate damage to the structure. A 4294 Agilent impedance analyzer was used to obtain measuring data, in two frequency ranges, 50–60 kHz and 110–120 kHz [12]. In such case, the major problem was the absence of a study about the effect of the temperature. In [5], there were experimental tests in tubular structures of different types and sizes of piezoelectric ceramics. For damage detection, two damage metric indices were used, the RMSD (Root Mean Square Deviation) and the CCDM (Correlation Coefficient Deviation Metric). The authors demonstrated that damage detection in tubular structures using low cost piezoelectric transducers is viable. In Ref. [4], the authors carried out a review of several damage detection methods based on guided waves and applied to different types of structures, including pipelines, showing the advancements in recent years. Generally speaking, some experiments present difficult replication, especially considering different shapes and sizes of the structures.

In EMI-based damage detection techniques, in a general way, it can be observed that the execution of experiments to demonstrate the efficacy of such techniques is somewhat limited due to some reasons. In the case of sensors, for example there is a variety of PZT types, with different compositions, geometry, and stiffness. In the case of structures, there may be some differences on the type of material, size, and geometry. In addition, to perform an experiment, it is still necessary to damage the structure or simulate damage by placing small screws on the structure, for example. Such combinations may difficult some types of experiments. Therefore, to simplify the study and development of SHM techniques based on EMI, numerical methods are considered. A variety of options become possible when using numerical methods, since frequency response functions (FRF), which correspond to the electrical signatures studied on the EMI technique, can be characterized and analyzed by means of modeling and simulation programs based on finite elements analysis. Thus, in many cases, commercial finite element (FE) modeling software packages are used to analyze and simulate SHM systems based on EMI [13]. This way, there are several studies that use numerical simulators based on FE, such as ANSYS, PZFlex, or ABAQUS, all applied to SHM systems.

From the moment that the numerical analysis began to be explored in the EMI context, many works have emerged. In Ref. [13], the authors modeled and analyzed a simple aluminum structure using ANSYS software, with variations in size and thickness of the sensors, and with different coupling to the structure (bonded or loose). Efficiency assessment was based on the analysis of the real part of the PZT impedance, which was considered efficient in detecting local damage. In Ref. [14], the authors used the software ABAQUS to model, via finite elements, underground oil pipelines and analyze the propagation characteristic of the guided waves generated by a PZT. Different types of damage were simulated, and the detection was performed using guided waves with a 70 kHz central frequency. The numerical results were compared to practical ones (measurements) and were considered satisfactory [15].

Despite many previous studies on monitoring the pipeline and ducts structural integrity using the EMI technique, there is not a definitive solution yet. The major problem is compensating the temperature variations, as they affect the transducers properties and hinder real applications. Thus, the environmental temperature variation is cited in the literature as a critical problem for applications based on EMI since small alterations in temperature may cause significant changes in the electrical impedance signature of PZT transducers, on the same level of small damage [16].

In this context, many authors have investigated the effects of temperature variation for detecting structural damage based on EMI and have proposed solutions to compensate such variations [9,16,17,18,19,20,21,22,23,24,25,26,27]. The most recurrent effects observed on EMI signatures are horizontal (frequency) and vertical (magnitude) shifts. Each technique proposed presents its advantages and limitations, which are present in the following.

Among the methods applied to compensate those shifts, there exist the ones based on effective frequency shift (EFS) and correlation analysis, and its variations [9,16,18,19,20,21]. The main limitation of this approach is that the EFS is constant over the frequency range; however, it was observed by some authors [9,16,19] that the frequency shift changes according to the frequency of the resonance peak analyzed, since it usually increases as the frequency range increases. Therefore, this proposal reaches suitable results for narrow frequency ranges and it loses efficiency as the frequency range increases due to the frequency shift not be constant over large frequency spectrums. To overcome this drawback, in [19], the entire compensation range was broken into small sub-bands. For compensating the vertical shifts, some of the EFS method usually incorporates a shift based on the difference of mean values of the signatures, with some variations [9,18,20,21]; however, when the temperature range becomes large, some authors considered the use of more than one baseline in order to have a more precise vertical compensation [9]. On other studies, normalization is used to reduce the vertical shift [19].

Ref. [22] uses Lagrange interpolating polynomials for generating a compensated baseline at a given temperature. First, a horizontal compensation was done by tracking the peak impedance value of the impedance range. Second, a 2nd degree Lagrange interpolation was used to compensate the vertical shifts. One advantage is that this approach accounts for nonlinear (quadratic) dependence of the vertical shifts; however, the horizontal shift applied appears to be similar to the EFS approach, which is effective only for narrow frequency ranges.

Ref. [23] proposed a normalized linear temperature-dependent coefficient for compensating the EMI signatures since the goal of the temperature compensation is to eliminate the temperature effect of the PZT sensor only, for a temperature range from 80°F to 160°F. This proposition does not eliminate the horizontal shift.

Other authors applied the linear principal component analysis (PCA) to filter out and remove the temperature effects on impedance signatures [17,25,26]. In Ref. [26], the damage detection in prestressed tendon anchorages was conducted by filtering impedance signatures; however, accuracy depends on the choice of the principal components, which are significantly influenced by the temperature variation. Overall, the study lacks investigations regarding wider temperature and frequency ranges.

In addition, several EMI damage detection systems based on pattern recognition algorithms have been developed using Artificial Neural Network (ANN) [28,29]. Moreover, among the ANN architectures, the radial basis function (RBF) is considered a powerful curve fitting tool [24]; thus, they have been applied for compensating the temperature effect in impedance-based SHM systems [24,27]. More specifically, Ref. [27] designed an RBF network-based regression algorithm for training a set of baseline measurements at various temperatures to compensate the temperature effect for each frequency sample of the EMI signatures. The authors also considered that the electrical impedance was a function of temperature and frequency; however, in this approach, it is not necessary to know how this dependence behaves (i.e., linear or quadratic). When using ANN, it is necessary to train the network in several different situations so that it can learn the pattern and present the correct result for untrained situations. In this study, to achieve accurate results, it was necessary the use of at least 168 training patterns. This is a large amount of pre-stored information, which is not always possible to obtain. Moreover, the computational load increases with the increase of the frequency range (number of frequency samples).

Nevertheless, considering that the accuracy on the temperature measurements is crucial when applying temperature compensation techniques and that the temperature on the structure could vary heterogeneously depending on its size or type of material, references [30,31] take advantage of the electrical impedance temperature dependence, so that the signatures themselves can be used for estimating the temperature of the PZT and structure, and for monitoring the soil freezing-thawing process, which is important for underground pipelines. By doing so, any temperature compensation technique becomes more economical since temperature sensors are not necessary anymore.

Moreover, the use of numerical methods and analytical models is becoming an important alternative for the study and development of temperature variation compensation methods since the temperature dependence of the PZT’s and structure’s properties can be analyzed separately [32,33]. 

On the basis of the previous works, the contributions of this paper lie in the following: First, this work outlines a complete analytical, numerical, and experimental study on the effect of the temperature on damage detection in pipelines, based on the EMI technique. Second, this study proposes an innovative approach for compensating the temperature effects on damage detection. The proposed method eliminates temperature effects through a simples and practical compensation method based on linear interpolation, which can be applied mainly to structures that present impedance amplitude and frequency shifts with linear dependence of the temperature and frequency. This paper introduces an algorithm with a very low computational complexity. The proposed method aims to use a minimum number of previously stored baselines. Third, this study examines two different damage metric indices (RMSD and CCDM indices) to evaluate damage detection in tubular structures. Finally, the feasibility of the proposed computational algorithm is verified by monitoring healthy and damaged structures under temperature-varying condition. The proposed method was tested in two steel pipes, compensating the temperature effect ranging from −40 °C to +80 °C, with analysis on the frequency range from 5 kHz to 120 kHz.

## 2. EMI Technique and the Effect of Temperature

The EMI technique is based on the piezoelectric effect, which allows establishing a relation between the mechanical properties of the host structure and the electrical impedance of the PZT transducer attached to the structure. This relation states that any change in the mechanical impedance of the structure will produce a variation in the electrical impedance measured through the PZT transducer. The electromechanical interaction of a PZT patch with its host structure is described by Equation (1) [8,20,23,34]:

(1) $$Y=Z^{-1}=i\omega .\frac{W_{a}l_{a}}{h_{a}}\left(\varepsilon_{33}^{T}\left(1-\delta i\right)-\frac{1}{1+\left(Z_{a}(\omega )/Z_{s}(\omega )\right)}.\;d_{3x}^{2}.\;Y_{xx}^{E}\right)$$

where *Y* is the electrical admittance (inverse of impedance *Z*), *Z*_a_ is the mechanical impedance of the PZT, *Z*_s_ is the mechanical impedance of the structure, $Y_{xx}^{E}$ is the Young’s modulus of the PZT at zero electric field (inverse of compliance), *d*_3x_ is the piezoelectric strain constant at zeros stress, $\varepsilon_{33}^{T}$ is the permittivity at zero stress, *W*_a_ is the width of the PZT, *l*_a_ is the length of the PZT, and *h*_a_ is the thickness of the PZT. The parameter *δ* is the dielectric loss factor. As mentioned before, temperature changes cause significant variations on the electrical impedance [16,23,32,35]. That occurs because the properties of the structure and the PZT, represented by the parameters in Equation (1), are affected by temperature [36,37].

### 2.1. Effect of Temperature on Parameters of the PZT

The piezoelectric ceramic parameters are highly dependent on temperature. The dielectric constant $\varepsilon_{33}^{T}$ is known to vary significantly with temperature [21,23]. Usually such dependence is nonlinear, but it may be characterized by a quadratic or cubic function. The Young’s modulus at zero electric field of PZT, $Y_{xx}^{E}$, is known to be slightly temperature dependent and it is affected by changes in the electric field [23,36]. Also, the piezoelectric coupling constant, *d*_3x_, in the arbitrary *x* direction at zero stress, is known to vary slightly with temperature. An increase in temperature leads to a very minimal increase in *d*_3x_ [23]. Due to the thermal expansion coefficient, the PZT dimension, represented by *W*_a_, *l*_a_, and *h*_a_, varies with temperature. Although such variations do affect the PZT operation, most parameters can be modeled as linearly temperature dependent.

To characterize the behavior of the resonance frequencies depending on the temperature, each of the parameters is represented by one type of function, linear or quadratic, considering the observed variation. Besides that, considering the complexity to represent impedances *Z*_a_ and *Z*_s_ on Equation (1) as a function of frequency and temperature, the authors use the following equivalent equation:

(2) $${\left[1+\left(Z_{a}(\omega )/Z_{s}(\omega )\right)\right]}^{-1}=\left(a_{1}+b_{1}\omega \right)\left(a_{2}+b_{2}T\right)$$

where *a*_1_, *b*_1_, *a*_2_, and *b*_2_ are only constants.

Deriving the expression proposed on Equation (2) in relation to *ω* and equaling to zero, the resonance frequencies are obtained. An approximation that specifies the frequencies of resonance as a function of the temperature, according to the requirements of Equation (2) is given by:

(3) $$\omega =a_{3}+b_{3}T$$

where *a*_3_ and *b*_3_ are only constants.

For a more precise analysis, real data obtained from each parameter would have to be considered, but unfortunately piezoelectric materials manufacturers do not provide such information. The linear temperature dependence of the resonance frequencies, Equation (3), will be evaluated when analyzing the experimental and simulated results.

In this work, for the development of a PZT/structure coupled system model considering the effect of temperature, it was necessary to characterize the main temperature-dependent parameters of both structure and PZT. To adequately model the piezoelectric materials, information on all its 13 independent parameters must be provided [38]. The values of the material’s coefficients in relation to temperature, for the EC-65 PZT (which is the equivalent of a PZT 5A), were obtained from [37]. Based on these results, the values of the following parameters were computed, ranging from −40 °C to +80 °C: $d_{15},d_{31},d_{33},S_{11}^{E},S_{12}^{E},S_{33}^{E},$ $S_{44}^{E},\varepsilon_{11}^{T}$ and $\varepsilon_{33}^{T}$. On the other hand, regarding the density and mechanical *Q* factor, their values were not characterized as a function of temperature, so the values provided by the manufacturer at room temperature were considered. The elasticity coefficient $S_{66}^{E}$ was calculated according to [39], as follows:

(4) $$S_{66}^{E}=2(S_{11}^{}-S_{12}^{})$$

### 2.2. Effect of Temperature on Parameters of the Structure

The second term of Equation (1) includes the mechanical impedance for both PZT (*Z*_a_) and host structure (*Z*_s_). When the PZT is bonded to the structure, *Z*_a_ is fixed and, therefore, *Z*_s_ exclusively determines the contribution of the second term for impedance final value. The contribution of the second term is shown in the spectrum of impedance as sharp peaks on the electrical impedance. Since such peaks correspond to a specific structural resonance, they constitute a unique description of the dynamic behavior of the structure [23]. Thus, considering that *Z*_s_ depends on the excitation frequency and temperature and that the Young’s modulus of the structure varies slightly in relation to the temperature [23,40], the effect of the variation of *Z*_s_ is an alteration of the resonance peaks, producing, especially, a frequency shift. Therefore, to take in account the temperature dependence of the structure, the density of the steel as a linear function of temperature and its thermal expansion coefficient, 12 × 10^−6^ °C^−1^, were used on the simulation.

### 2.3. Correlation Coefficients to Compare Simulated and Experimental Responses

To validate the electrical impedance measurements, the coefficient of correlation of Pearson (*C*_C_) was used in both experiment and simulation. The coefficient of correlation of Pearson (*C*_C_) is usually used to measure the degree of correlation between two variables of metric scale. Thus, Equation (5) is used to assess the degree of correlation between the two electrical impedance signatures *Z*_E_ and *Z*_S_, obtained through simulation and experimentally, respectively. ${\bar{Z}}_{S}$ and ${\bar{Z}}_{E}$ are the average values of these signatures for the selected frequency range ($\omega_{I}$ to $\omega_{F}$).

(5) $$Cc=\left|\frac{\sum_{k=\omega_{I}}^{\omega_{F}}\left[Z_{S}(k)-\bar{Z_{S}}\right]\;\left[Z_{E}(k)-\bar{Z_{E}}\right]}{{\sum_{k=\omega_{I}}^{\omega_{F}}\left[Z_{S}(k)-\bar{Z_{S}}\right]}^{2}\;{\sum_{k=\omega_{I}}^{\omega_{F}}\left[Z_{E}(k)-\bar{Z_{E}}\right]}^{2}}\right|$$

On the other hand, there is the coefficient of correlation of Spearman, which is also used to compare and validate two results. Such coefficient, frequently described as “non-parametric”, is generally used to relate linear and nonlinear systems. A perfect Spearman correlation occurs when the variables *Z*_E_ and *Z*_S_ are related to any monotonous function, in contrast to Pearson’s correlation, which gives a perfect correlation when *Z*_E_ and *Z*_S_ are related by a linear function. Thus, Equation (6) is used to assess the coefficient of Spearman.

(6) $$r_{s}=1-\frac{6\sum_{i-1}^{n}{\left[Z_{E}(i)-Z_{S}(k)\right]}^{2}}{n(n^{2}-1)}$$

When applying Equations (5) or (6), an analysis of the coefficient of correlation is done, *C*_C_ or *r*_s_. To obtain a very strong positive correlation, the value must be between 0.80 and 1.00 [41].

## 3. Simulations and Experimental Validation

Modeling through finite elements analysis (FEA) allows computationally assessing how the system reacts to real-world stimuli, such as vibration, temperature, fluid flow, and other physical effects. To do so, there exist a great number of software packages, such as ANSYS, Atila, Comsol, PZFlex, ABAQUS, etc. The PZFlex^®^ is a commercial software package for FEA and virtual prototyping, focused on time domain analysis and wave propagation problems, with emphasis on electromechanical materials, such as piezoelectric ceramics [42]. However, the application of a software to implement a FEA model requires detailed knowledge of the system and its parameters to be modeled. 

Focusing on efficiency and availability, in the present work the PZFlex was used to model, simulate and study the effect of the temperature variation in steel-pipe/PZT coupled systems, with or without damage, based on the EMI technique. Computational algorithms were developed to model and execute simulations, recursively adjusting the values of the parameters dependent on the temperature, previously calculated for the range from −40 °C to +80 °C. After each simulation, a PZT electrical impedance spectrum is obtained and, next, the temperature is changed. 

### 3.1. Experimental Setup and Simulation Models

**Figure 1:**
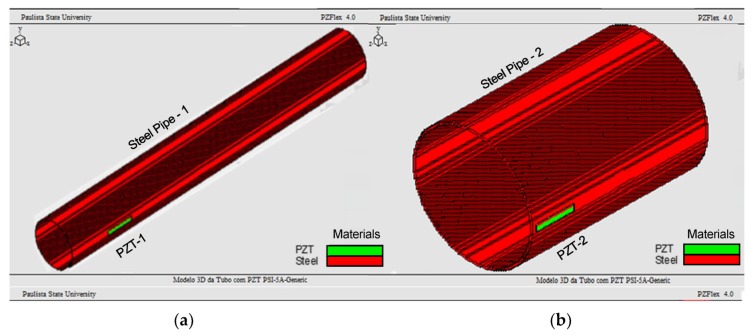
Models of steel- pipe/PZT systems: (**a**) pipe-1 and (**b**) pipe-2.

**Figure 2:**
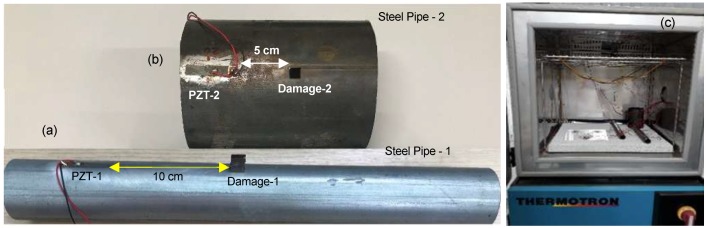
Steel- pipe/PZT real systems: (**a**) pipe-1; (**b**) pipe-2; and (**c**) Environmental chamber.

Two steel pipes of different sizes with PZTs patches attached to were modeled and simulated using PZFlex software, as shown in Figure 1. Both simulated structures were based on the real structures pipe/PZT indicated in Figure 2. In the software, only healthy structures were modeled and simulated; in the experiments, however, healthy and damaged conditions were tested. Damage was generated in each structure by attaching masses to their external surface. Figure 2 shows the PZTs and damage bonded to each structure as well as the damage locations. The dimensions and characteristics of the materials employed in the simulations and experiments are described in Table 1.

To simulate the models under the effect of temperature variation and calculate the electrical impedance signatures of the PZTs bonded to the pipes, the PZT and pipe parameters shown in Table 2 were characterized.

To measure the electrical impedance of the PZTs bonded to the pipes, under the effect of temperature variation, experiments were performed in the Laboratory of Control and Intelligent Systems of the Department of Mechanical Engineering of UNESP/Ilha Solteira. The specimens under test (PZT/Pipes, Figure 2c) were placed in a SM-8-8200 THERMOTRON climatic chamber. The measurements were obtained using an impedance analyzer developed by [43], and a USB-6259 data acquisition system from National Instruments. Measurements for the baseline conditions (healthy pipes) and with simulated damage, were done for both specimens, varying the temperature from −40 °C to +80 °C, with steps of +10 °C, for the frequency range from 0 to 125 kHz.

### 3.2. Correlation Between Experimental and Simulated Electrical Impedance Signatures

**Figure 3:**
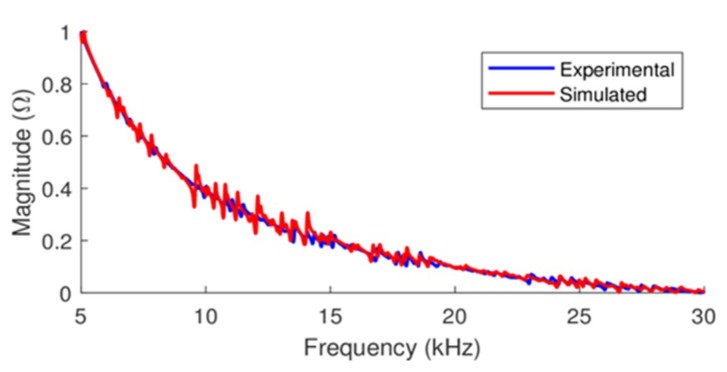
Normalized electrical impedance signatures for pipe-1/PZT-1: experimental and simulated.

The validation of a numerical model requires a comparative analysis between experimental results and simulated results and, for that, pipe-1/PZT-1 was used as reference. Figure 3 presents the normalized signatures of electrical impedances obtained experimentally and through a numerical model implemented using PZFlex, for frequency range from 5 to 30 kHz, since it is the most sensitive range. The electrical impedances shown in Figure 3 correspond to PZT-1 bonded to pipe-1 and were obtained at +20 °C.

To evaluate the correlation degree between the experimental and simulated signatures of the electrical impedance (magnitude), as shown in Figure 3, Equations (5) and (6) were used. The values calculated for the correlation coefficients of Pearson and Spearman, are 0.9968 and 0.9945, respectively. As previously stated, these values indicate the existence of a very strong correlation between the analyzed variables.

Therefore, considering that exist a very strong correlation between experimental and simulated results, it can be concluded that the implemented models “faithfully” represent the behavior of real structures for purposes of studying the EMI technique, even considering the small discrepancies observed in Figure 3. Thus, in the following sections, the results of the simulations of the pipe-1/PZT-1 system under the effect of temperature variation obtained using PZFlex will be analyzed.

## 4. Analysis of the Effect of Temperature Variation Applied to Steel Pipes

**Figure 4:**
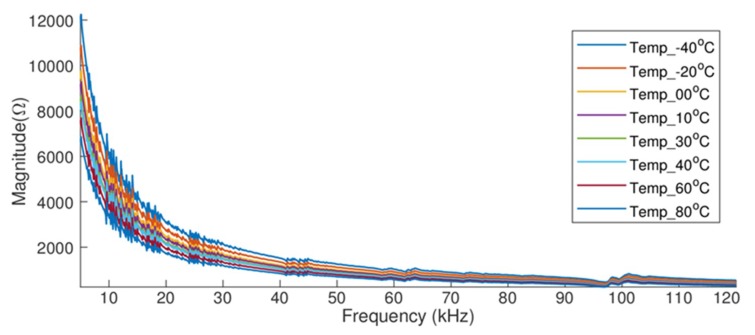
pipe-1/PZT-1 electrical impedance signatures under the effect of temperature.

The system model composed of the PZT-1 attached to pipe-1, which was simulated and validated in Section 3, is also used in this section to study and analyze the effect of temperature variation in steel pipes. The characteristics of the PZT-1 and pipe-1 and the temperature-dependent parameters included on the simulation were detailed in Table 1 and Table 2, respectively (Section 3). Figure 4 shows the numerical electrical impedance signatures obtained from pipe-1/PZT-1 for different temperatures, ranging from −40 °C to +80 °C and for the frequency range from 5 kHz to 125 kHz. As already mentioned, the results confirm that the electrical impedance signatures shift in frequency and magnitude as temperature changes.

To study and understand such effects in a more detailed way, results from three numerical analysis corresponding to three different cases are analyzed and discussed in the following sections. For each case, the model is simulated changing the parameters’ values of each material (PZT-1 and pipe-1), separately and together. For more details and better evaluation, results are presented for only two narrow frequency ranges: 8.6 kHz to 19.2 kHz and 96 kHz to 106 kHz.

### 4.1. Case 1: Changes in the Steel Pipe Properties as a Function of Temperature Variation

**Figure 5:**
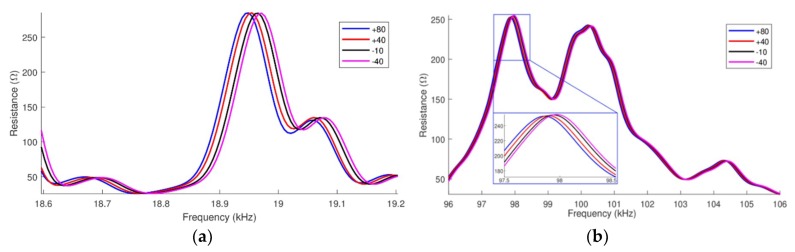
Horizontal shift of the impedances (real part) pipe-1/PZT-1: (**a**) at low frequency and (**b**) at high frequency.

In the first case, only the temperature-dependent parameters of the pipe-1 were changed. Figure 5 presents the signatures of the electrical impedances (real parts) for the two narrow frequency ranges analyzed. In both Figure 5a,b, it is possible to observe that the main effect is a horizontal shift to the left as the temperature rises. For the peaks located at “low frequency”, plotted between 18.9 kHz and 19 kHz in Figure 5a, the horizontal shift between the signatures measured at −40 °C and +80 °C is 20 Hz. However, for the “high frequency”, which means for peaks plotted around 98kHz in Figure 5b, the horizontal shift is 106 kHz.

Thus, as it was expected, the main effect of the temperature in the pipe parameters is a frequency shift on the electrical impedance signatures. It is also noticed that as the frequency increases, the horizontal shift also increases. Besides that, it can be observed that there is a small change in the amplitude of the electrical impedance when the temperature varies.

### 4.2. Case 2: Changes in the PZT Properties as a Function of Temperature Variation

**Figure 6:**
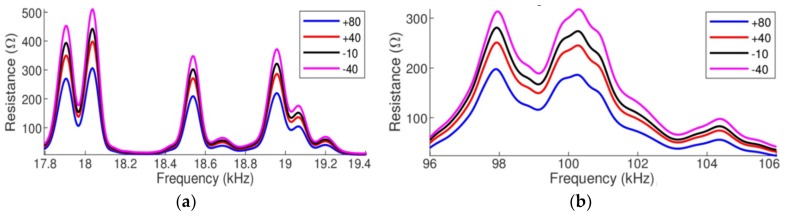
Vertical shift of the impedances (real part) pipe-1/PZT-1: (**a**) at low frequency and (**b**) at high frequency.

In the second case, for each simulation, only the PZT-1 temperature-dependent parameters were changed. Figure 6 presents the signatures of the electrical impedance (real part), for the same two narrow ranges. It can be observed in both frequency ranges that the amplitude decreases (vertical shift) as the temperature increases and such shift is more expressive at the resonance peaks. However, as in the first case, there is also a horizontal shift that cannot be disregarded.

Therefore, the conclusion is that the main effect of temperature variation in the PZT parameters is a vertical shift in the impedance amplitude.

### 4.3. Case 3: Changes in the Properties of both PZT and Structure as a Function Temperature Variation

**Figure 7:**
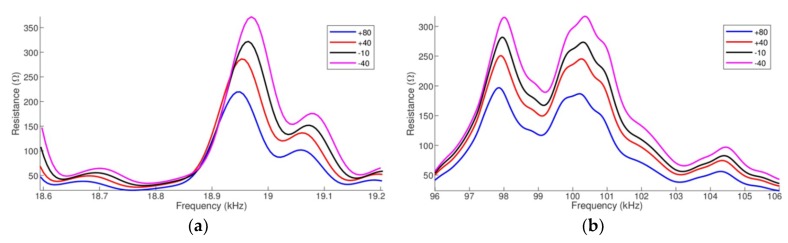
Horizontal and vertical shifts of the impedances (real part) pipe-1/PZT-1: (**a**) at low frequency and (**b**) at high frequency.

In the third case, for each simulation the temperature-dependent parameters of both PZT-1 and pipe-1 were changed. Figure 7 shows the signature of the real part of the electrical impedance for the same two narrow ranges. In this case, as shown in Table 3, there are both horizontal/vertical shifts of 22 Hz/152.35 Ω in “low frequency” and both horizontal/vertical shifts of 155 Hz/118.20 Ω in “high frequency”, between the signature’s peaks at −40 °C and 80 °C.

Table 3 presents a summary of the effects of temperature variation in the three cases. The values of Case 3 indicate that there is a superposition of both effects corresponding to Cases 1 and 2.

The results clearly show a direct relation between the horizontal shifts and the thermal expansion coefficient of the host structure, as demonstrated by the first case. The horizontal shifts increase as the frequency does. There is evidence that the amplitude shifts are present when parameters of piezoelectric material are also changed depending on the temperature, as shown in the second case. Thus, in the third case, both effects can be simultaneously observed. However, based on the results of Table 3, it is concluded that the PZT parameters have also influenced, in a lower degree, on the horizontal shift. Notwithstanding, the parameters of the structure, which are temperature dependent, practically do not have much influence in the vertical shifts. 

The same effects observed in Section 4.1, Section 4.2 and Section 4.3 were reached by the analytical temperature-dependent model proposed in [32] and by the temperature-dependent spectral element model (SEM) model from [33] when the properties of both PZT and structure were analyzed separately and together.

To develop and apply a compensation mechanism of the temperature effect, it is necessary to know how the trendlines of the horizontal and vertical shifts would be. The following section studies 3D graphic for a better understanding of these trendlines.

### 4.4. Analysis of the Impedance Signatures Behavior Through 3D Graphics

**Figure 8:**
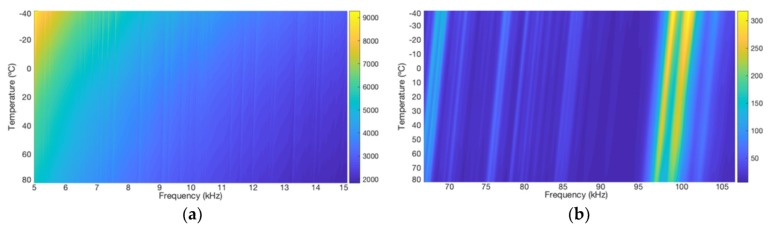
Trendlines of the horizontal shift of the impedance magnitude pipe-1/PZT-1: (**a**) at low frequency and (**b**) at high frequency.

**Figure 9:**
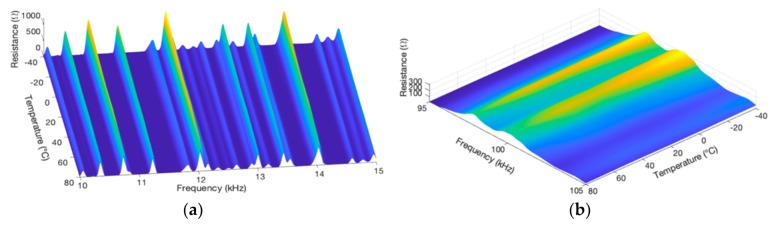
Trendlines of the vertical shift of the impedance (real part) pipe-1/PZT-1: (**a**) at low frequency and (**b**) at high frequency.

To analyze the spatial behavior of the electrical impedance measurements depending on the temperature in a more detailed way, 3D graphics were plotted. Figure 8 presents two separate views of a 3D graphic of the impedance magnitude, one view for low frequency and the other for high frequency. The magnitude values are represented by a color bar. Figure 9 presents two separate views of a 3D graphic of the real part, also for two narrow frequency ranges. 

In Figure 8, analyzing the amplitude of the resonance peaks that stand out by the lighter colors, it is possible to observe that as the temperature changes, the peaks present frequency shifts with linear trendlines but which are not parallel throughout the entire range. The trendlines in Figure 8, for low frequencies, are more vertical, while in Figure 8b, for high frequencies, the trendlines are more tilted. The conclusion is that the inclinations of trendlines, in relation to the frequency axis, decrease as the frequency increases.

Moreover, as mentioned, by looking at Figure 8 it is observed that the peaks present linear frequency shifts as the temperature changes. This behavior reinforces the approximation made in Equation (3), which specifies the frequencies of resonance as a linear function of the temperature.

Similarly, in Figure 9, analyzing the amplitude of resonance peaks, it can be verified that as the temperature changes, the trendlines are practically linear; however, the inclinations vary and depend on the peaks’ amplitude.

Finally, considering the simulation results for the pipe1/PZT-1, to propose new techniques to compensate the temperature effect, it can be concluded that the frequency shift shows a linear dependency, both depending on the temperature and frequency. Concerning the vertical shift, the amplitude changes are approximately linear depending on the temperature change.

## 5. Effect of Temperature on Impedance-Based Damage Detection

In the EMI technique, two electrical impedance signatures are compared to detect changes in the dynamic response of the structure. A monitoring signature is compared to a baseline signature, previously registered as a healthy structure. In the occurrence of damage, the monitoring signature will exhibit horizontal and vertical shifts in relation to the baseline signature. However, according to the literature and to the analysis presented in this work, the damage detection systems based on EMI present a strong dependence on temperature, and, therefore, any change associated with a temperature variation can be confused with damage, which means the detection of a false positive. 

For comparing both signatures, there are statistic indices or damage metric indices, normally based on the CCDM and RMSD. Overall, the CCDM index is more sensitive to changes in the shape of the impedance signature and the RMSD is more sensitive to variations in amplitude of the impedance signature. The smaller the metric values, the “closer” are the signatures [23]. 

**Figure 10:**
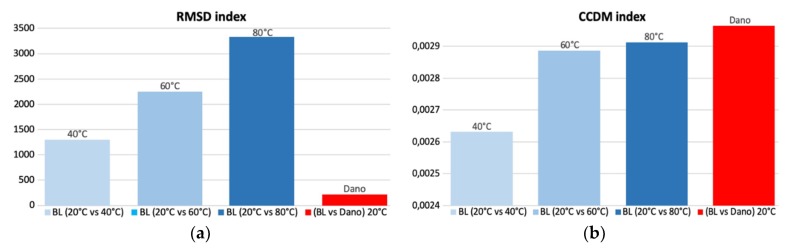
Damage metrics and the effect of temperature pipe-1/PZT-1: (**a**) RMSD and (**b**) CCDM.

In this section, to calculate the CCDM and RMSD indices, the monitoring and baseline signatures obtained experimentally for pipe-1/PZT-1 were compared. The reference baseline signature was measured at +20 °C. The monitoring signatures were measured in two different situations. First, three baseline signatures were obtained only changing the temperature to +40 °C, +60 °C and +80 °C. Second, with the damage-1 attached to the structure, the monitoring signature was also measured at +20 °C. Figure 10 shows the indices calculated for the frequency range from 5 kHz to 40 kHz.

In Figure 10, it is observed that the real damage (red bar) presented a RMSD value lower than the values obtained by only changing the temperature, and a CCDM value very close to those obtained due to the temperature variation. In other words, in a real scenario, the temperature variation can cover real damage and it can also indicate false damage. The values of the indices get higher as the temperature increases. The same was observed when the temperature decreases, with the most significant differences registered for temperatures under 0 °C.

Therefore, for the EMI technique to be effectively employed, it is extremely required that the SHM systems include techniques or mechanisms to compensate the effects of temperature variation in the electrical impedance measurements from PZT sensors. In the following section, two mechanisms to perform the compensation of the temperature effect are presented.

## 6. Compensation of Temperature Effect in Impedance-Based SHM Systems

As exposed in the literature, several researchers have proposed solutions to compensate the temperature effect, but each of them indicates some restrictions, such as limitation for frequency and temperature ranges, the need for a large number of signatures and, in some cases, algorithms with high computational load. In this sense, the main challenge is still the search for new models to effectively compensate the temperature effect over wide frequency and temperature ranges. 

Thus, considering the discussions of Section 4 and Section 5, in this section, two algorithms to compensate temperature effects are presented. Based on all numerical analysis, the vertical shifts are assumed to be linearly dependent of temperature and the horizontal shifts are assumed to be linearly dependent of temperature and frequency. It is known that these dependences could be nonlinear for other structures; however, since for the simulated structures the linear dependence was observed, the linear compensation method explained in this section was established. The proposal also considers that in fact, there are structures or systems, such as aircraft for instance that operate in extreme temperatures. 

For the application of the method, at least two reference baseline signatures measured at different temperatures (*BL*_T1_ and *BL*_T2_) should be registered and stored before monitoring the structure. The method consists of estimating, based on a reference baseline, a new baseline (*BL*_comp_) for the temperature of operation (*T*_op_), in which the monitoring will be performed. First, one of the reference signatures is compensated in frequency until reaching the temperature of operation. Next, the compensation in amplitude is made. 

In the following sections, two algorithms to compensate the temperature effect are presented: the first one is to compensate the frequency shifts (horizontal compensation), while the second compensates for the amplitude variations (vertical compensation).

### 6.1. Algorithm to Compensate the Frequency Shifts: Horizontal Compensation

From the conclusions of Section 4.1, Section 4.3 and Section 4.4, two important aspects that aim at properly compensating the frequency shift are highlighted. The first is that the electrical impedance signature suffers a horizontal shift to the left as the temperature increases and vice-versa as shown in Figure 7. The second is that the impedance values range throughout a linear trendline as the temperature changes, but the inclination depends on the frequency. In this case, the higher the frequency, the smaller the inclination, as shown in Figure 8. 

**Figure 11:**
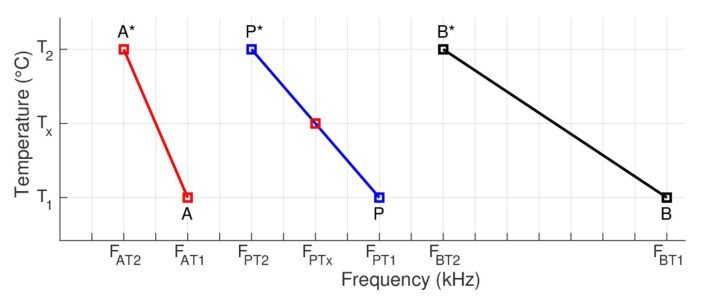
Geometric representation of frequency shifts.

Therefore, Figure 11 illustrates the information of two resonance peaks, identified as *A* and *B* for temperature *T*_1_ and, identified as A* and B* for temperature *T*_2_. These peaks should correspond to two reference baselines, known and measured at temperatures *T*_1_ and *T*_2_. In the figure, *F*_AT1_ and *F*_AT2_ are the frequencies of peak *A* obtained at temperatures *T*_1_ and *T*_2_, respectively, while *F*_BT1_ and *F*_BT2_ are the frequencies of peak *B* obtained at temperatures *T*_1_ and *T*_2_, respectively.

Thus, the goal is to calculate the frequency shift and determine the new *F*_PTx_, frequency of a given impedance measurement *P*, measured at the temperature *T*_x_. In the method, once *P* is chosen, first, the frequency of P* should be calculated. In this case, *P* corresponds to a signature obtained at *T*_1_ and P* a signature obtained at *T*_2_, where *F*_PT1_ is the frequency of *P* obtained at temperature *T*_1_ and *F*_PTx_ is the frequency that would correspond to *P* at temperature *T*_x_. Then, according to Figure 11, while the frequency of *P* might vary between the frequencies of *A* and *B* for the temperature *T*_1_, the frequency of P* may vary between the frequencies of A* and B* for the temperature *T*_2_. 

For this, Equation (7) defines the proportion between the frequency shifts from *A* to *P* and from *A* to *B*. 

(7) $$\frac{F_{PT2}-F_{AT2}}{F_{BT2}-F_{AT2}}=\frac{F_{PT1}-F_{AT1}}{F_{BT1}-F_{AT1}}$$

From Equation (7) and isolating *F*_PT2_, in Equation (8) the frequency of *P* can be calculated for the temperature *T*_2_.

(8) $$F_{PT2}=F_{AT2}+(F_{BT2}-F_{AT2}).\frac{\left(F_{PT1}-F_{AT1}\right)}{\left(F_{BT1}-F_{AT1}\right)}$$

In the triangle-rectangle that has as hypotenuse *P* − P*, the equivalences presented in Equations (9) can be similarly obtained:

(9) $$\frac{F_{PT1}-F_{PTx}}{F_{PT1}-F_{PT2}}=\frac{T_{X}-T_{1}}{T_{2}-T_{1}}\;\mathrm{or}\;\frac{F_{PTx}-F_{PT2}}{F_{PT1}-F_{PT2}}=\frac{T_{2}-T_{X}}{T_{2}-T_{1}}$$

Therefore, based on (9), isolating *F*_PTx_, in (10) there are two alternatives to calculate the frequency of *P* for the temperature of the operation *T*_op_ = *T*_X_.

(10) $$F_{PTx}=F_{PT1}-(F_{PT1}-F_{PT2}).\frac{\left(T_{X}-T_{1}\right)}{\left(T_{2}-T_{1}\right)}\;\mathrm{or}\;F_{PTx}=F_{PT2}-(F_{PT1}-F_{PT2}).\frac{\left(T_{X}-T_{2}\right)}{\left(T_{2}-T_{1}\right)}$$

The use of the first expression (left side) is recommended if the temperature *T*_1_ is closer to *T*_op_. However, if the temperature *T*_2_ is closer to *T*_op_, it is recommended using the second expression (right side). The process is iterative; therefore, it must be repeated for all frequency samples, in other words, the impedance measurements that are intended to compensate. Nevertheless, it is clear that the process of horizontal compensation depends on the frequency.

### 6.2. Algorithm to Compensate the Amplitude Shift: Vertical Compensation

Based on the finding of Section 4.2, Section 4.3 and Section 4.4, to compensate the amplitude shift, there are two hypotheses. The first is that the impedance amplitudes vary linearly with temperature, since, as the temperature increases, the amplitude decreases following a linear trendline. The second is that the higher the amplitude, the bigger the vertical shift. Therefore, for the amplitude compensation, a shift is applied for each frequency sample, depending on the temperature and on the amplitude of both reference baseline signatures aligned in frequency. For this reason, the horizontal compensation must be performed first. 

**Figure 12:**
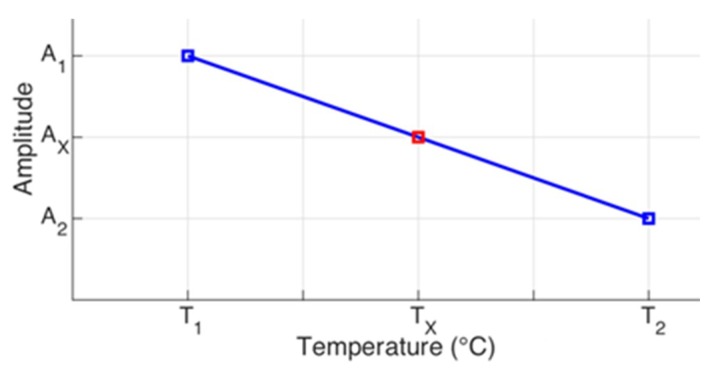
Geometric representation of the amplitude shifts.

Then, Figure 12 illustrates the information of just one resonance peak, identified as *A*_1_ and *A*_2_, which corresponds to the reference baseline signatures, registered at temperatures *T*_1_ and *T*_2_, respectively. Before applying the method, the reference baseline signatures should be aligned in frequency, in other words, the frequencies of *A*_1_ and *A*_2_ should be the same. This way, for the first hypothesis, *A*_1_ and *A*_2_ belong to the same line. Therefore, for the same frequency sample, the (*A*_x_) amplitude for any operation temperature (*T*_x_) will be also located at the straight line.

Applying the similarity of triangles, Equation (11) is defined, in two ways, as the proportion between the amplitude shifts as a function of temperature.

(11) $$\frac{A_{1}-A_{X}}{T_{X}-T_{1}}=\frac{A_{1}-A_{2}}{T_{2}-T_{1}}\;\mathrm{or}\;\frac{A_{X}-A_{2}}{T_{2}-T_{X}}=\frac{A_{1}-A_{2}}{T_{2}-T_{1}}$$

From Equation (11), isolating *A*_X_, in Equation (12) there are two alternatives to calculate the amplitude for the temperature of operation *T*_op_ = *T*_X_.

(12) $$A_{X}=A_{1}+(A_{1}-A_{2}).\frac{\left(T_{1}-T_{X}\right)}{\left(T_{2}-T_{1}\right)}\;\mathrm{or}\;A_{X}=A_{2}+(A_{1}-A_{2}).\frac{\left(T_{2}-T_{X}\right)}{\left(T_{2}-T_{1}\right)}$$

In this case, it is also recommended using the first expression (left side), if the temperature *T*_1_ is closer to *T*_op_. However, if the temperature *T*_2_ is closer to *T*_op_, it is recommended using the second expression (right side). The process is iterative and should be repeated for all frequency samples, i.e., the impedance measurements that are intended to compensate.

## 7. Results and Discussions

This section presents the results of the compensation method applied to two PZT/pipe structures presented in Section 3.1. The proposed method was tested using the electrical impedance signatures obtained experimentally and through simulation. Damage detection was performed using metrics based on CCDM and RMSD indices. For the results obtained with the PZFlex simulator, in the cases analyzed in Section 4, the compensation of the temperature effect using two algorithms were tested. In Section 7.1, the horizontal shifts observed on Case 1 and Case 3 are compensated, while in Section 7.2, the vertical shifts observed on Case 3 are compensated.

The complete method (horizontal and vertical compensation) was also applied to the experimental results obtained for the pipe-1/PZT-1 and pipe-2/PZT-2. The results of the compensations are presented in Section 7.2; Section 7.3 includes the damage detection evaluation by the RMSD and CCDM indices. 

### 7.1. Horizontal Compensation

**Figure 13:**
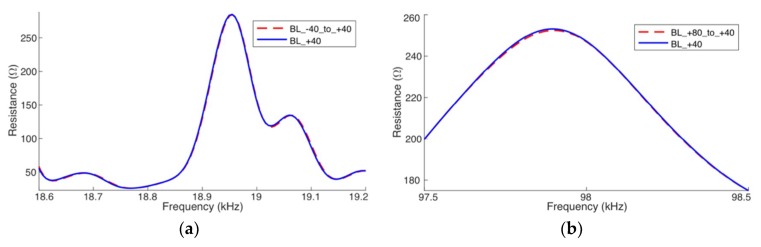
Horizontal compensation—Case 1 (Simulation) pipe-1/PZT-1: (**a**) at low frequency and (**b**) at high frequency.

**Figure 14:**
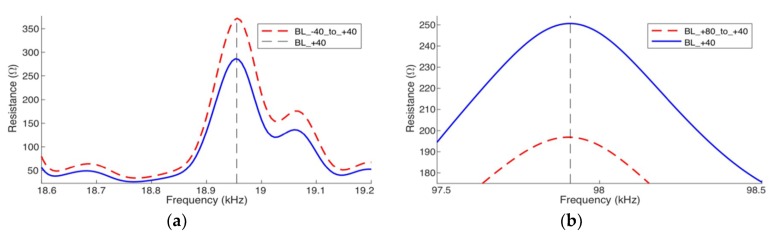
Horizontal compensation—Case 3 (Simulation) pipe-1/PZT-1: (**a**) at low frequency and (**b**) at high frequency.

Figure 13 and Figure 14 show the results obtained after compensating the frequency shift of the impedance (real part), from baselines calculated by the simulator. For a better visualization, the results are only presented for narrow frequency ranges; the left-side figures for low frequency and right-side figures for high frequency. Figure 13 shows the results obtained for Case 1 and Figure 14 for Case 3. For the compensations exhibited in Figure 13 and Figure 14, the reference baselines (*BL*_T1_ and *BL*_T2_) were obtained at −40 °C and +80 °C, respectively. 

In Figure 13a and Figure 14a, the baseline obtained at −40 °C was horizontally compensated for +40 °C (red line). On the other hand, in Figure 13b and Figure 14b, the baseline obtained at +80 °C was compensated for +40 °C (red line). In all cases, the compensated signatures practically coincide with the baseline obtained at +40 °C (blue line), which indicated that the horizontal compensation method works.

**Figure 15:**
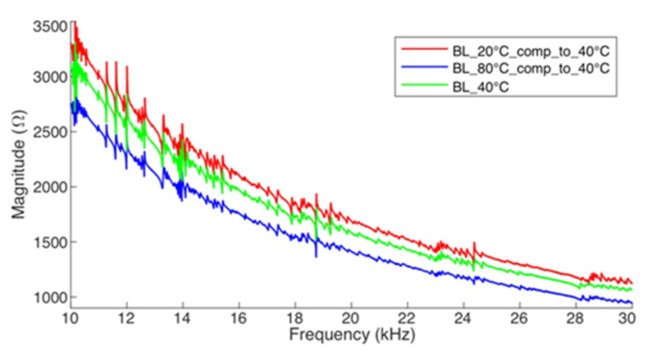
Horizontal compensation for pipe-1/PZT-1 (Experiment).

To check the horizontal compensation model with experimental results obtained for the pipe-1, Figure 15 shows electrical impedance baselines (magnitude) obtained at +20 °C (*BL*_T1_) and +80 °C (*BL*_T2_) horizontally compensated to +40 °C, i.e., both signatures are aligned with the frequency of the baseline measured at +40 °C. This important step allows estimating the signature compensated in amplitude for the operation temperature (*T*_op_ = +40 °C). Thus, for each frequency sample, Equation (12) can be applied, estimating new elements of compensated signatures from the reference baselines of *BL*_T1_ and *BL*_T2_. It is easily verified that the peaks of the compensated impedances are aligned to peaks of the baseline measured at +40 °C (green line).

### 7.2. Complete Compensation: Horizontal + Vertical

**Figure 16:**
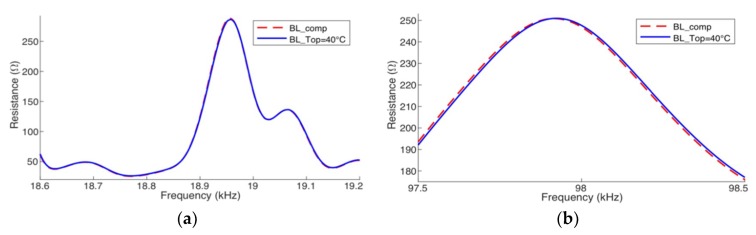
Complete compensation from +20 °C to +40 °C—Case 3 (Simulation) pipe-1/PZT-1: (**a**) at low frequency and (**b**) at high frequency.

Figure 16 presents, only for two narrow frequency ranges, the compensated results obtained for the real part of the impedance, from baselines calculated by the simulator in Case 3. The reference baselines *BL*_T1_ and *BL*_T2_ were obtained at +20 °C and +80 °C, respectively. The signature was compensated for *T*_op_ = +40 °C from *BL*_T1_. To check the efficacy of the compensation, the baselines obtained for the same *T*_op_ (blue line) are presented.

**Figure 17:**
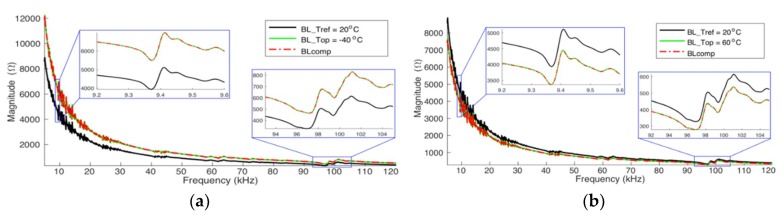
Complete compensation—Case 3 (Simulation) pipe-1/PZT-1: (**a**) from +20 °C to −40 °C and (**b**) from +20 °C to +60 °C.

Figure 17 presents the results for the complete range from 5 kHz to 120 kHz, attesting that the method works for wide ranges. The figure shows the signatures compensated for *T*_op_ = −40 °C and *T*_op_ = +60 °C from a reference baseline obtained at +20 °C. Thus, for both cases, *BL*_T1_ was obtained at +20 °C and *BL*_T2_ was obtained at +80 °C. Therefore, to qualitatively validate the performance of the proposed method, once again, it is noticed that when compared to the baselines obtained at the same *T*_op_ (green line), both signatures are extremely similar.

**Figure 18:**
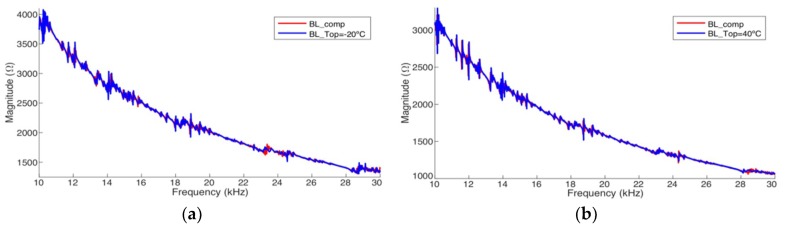
Complete compensation for pipe-1/PZT-1 (Experiment): (**a**) from −40 °C to −20 °C and (**b**) from +80 °C to +40 °C.

**Figure 19:**
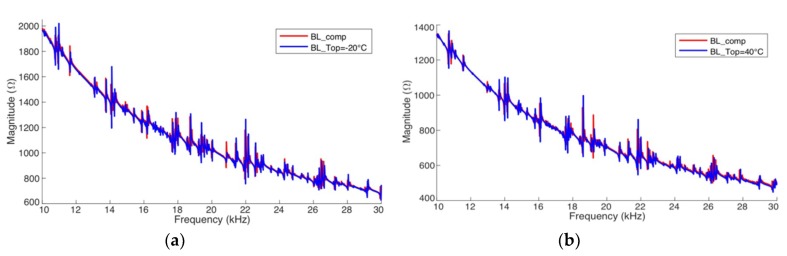
Complete compensation for pipe-2/PZT-2 (Experiment): (**a**) from −40 °C to −20 °C and (**b**) from +80 °C to +40 °C.

Figure 18 and Figure 19 present the results after the complete compensation of the experimental results of pipe-1 and pipe-2, respectively. The frequency range from 10 kHz to 30 kHz was chosen because this range presents the biggest resonances peaks, facilitating the assessment. For the compensations exhibited in these figures, *BL*_T1_ and *BL*_T2_ were obtained at −40 °C and +80 °C, respectively. Figure 18a and Figure 19a have compensated signatures for *T*_op_ = −20 °C from *BL*_T1_. Figure 18b and Figure 19b have compensated signatures for *T*_op_ = +40 °C from *BL*_T2_. 

The results show the efficacy of the compensation method, when applied to real pipe structures. Compensated signatures are very close to the baselines obtained at the same temperature of operation. However, it is noticed that there are peaks in the signatures showing notorious differences. This can be explained by the fact that there are secondary effects on the experimental measurements (instruments, materials and accessories), which are not observed in the results presented by the simulator (Figure 17).

### 7.3. Damage Detection Under the Effect of Temperature Variation

Monitoring a structure to detect damage with the EMI technique, without applying any temperature effect compensation method is generally performed through the CCDM and RMSD indices, which are calculated by comparing both monitoring and baseline signatures. The monitoring signature is obtained at the temperature of operation (*T*_op_), while the baseline is typically registered at room temperature for a healthy structure. However, to avoid false positives, in real applications, it is necessary to compensate the baseline for *T*_op_ before calculating the indices. In this way, this section presents some results of damage detection under temperature effect for the results obtained on the experiments. 

Firstly, the monitoring is performed in the healthy pipe-1/PZT-1 structure, only under the effect of temperature variation. Then, the monitoring is performed after sticking mass on the structure (damage-1 indicated in Table 1, Section 3.1), also under the effect of temperature variation. All the monitoring is performed at the temperature of operation (*T*_op_). For the compensation, from the reference baseline signature (*BL*_ref_), the compensated signature (*BL*_comp_) must be generated for the *T*_op_ temperature. In this case, the *BL*_ref_ was collected at *T*_ref_ = +20 °C, which is close to room temperature, but it could be measured at any other temperature. However, to improve the efficacy of the method, the use of a registered baseline at a temperature closer to *T*_op_ is recommended.

**Figure 20:**
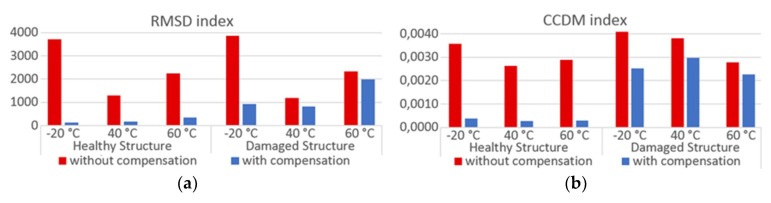
Damage index to assess the pipe-1/PZT-1 structural condition—Experiment: (**a**) without damage and (**b**) with damage.

**Figure 21:**
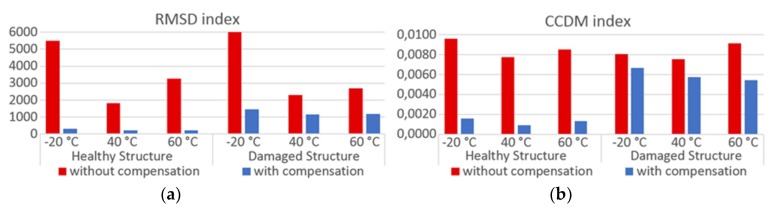
Damage index to assess the pipe-2/PZT-2 structural condition—Experiment: (**a**) without damage and (**b**) with damage.

To assess the structural condition of pipe-1/PZT-1 and pipe-2/PZT-2, the CCDM and RMSD indices are calculated comparing the baseline *BL*_comp_ to the monitoring signature. The indices presented in Figure 20 and Figure 21 were calculated for the frequency range from 10 kHz to 30 kHz and for different temperatures of operation −20 °C, +40 °C and +60 °C. Part (a) presents the indices for the healthy structure, only under the temperature effect; part (b) has the indices for the damaged structure and under the temperature effect. The red bars indicate indices calculated without compensation, in which the baseline signature corresponded to *BL*_ref;_ the blue ones indicate indices calculated after compensating the temperature effect, in which the baseline signature corresponded to *BL*_comp_. When applying the compensation for each *T*_op_ analyzed in Figure 20 and Figure 21, for *T*_op_ = −20 °C, *BL*_T1_ was measured at −40 °C and *BL*_T2_ was measured at +20 °C, while for *T*_op_ = +40 °C and *T*_op_ = +60 °C, *BL*_T1_ was measured at +20 °C and *BL*_T2_ was measured at +80 °C. 

The results in red shown in Figure 20 and Figure 21 indicate that the values of the indices calculated for the healthy structures without applying the compensation method are comparable to those obtained for the damaged structure, which would make any damage detection in a SHM system impossible. However, the results were different when the monitoring was made after applying the compensation method. In this case, the values of indices, for a healthy structure, are considerably smaller than for the damaged structure, even though some peaks in the signatures show notorious differences, as already mentioned. This proves the performance of the proposed method, making it possible to define a threshold to identify when one structure is damaged or when there is only variation in temperature. It should be highlighted that the CCDM index is more sensitive to damage, as expected.

Another point that should be highlighted is that the proposed method aims to use a small number of baselines previously stored, since it is difficult to obtain a large set of signatures. It contrasts to other methods, such as those based on radial basis function network (RBFN), where it is necessary to collect many signatures to train the network and obtain satisfactory results [27]. However, if it is possible to collect a vast number of baselines at different temperatures for structures with nonlinear dependences, it is suggested the evaluation of the linear method proposed, due to its simplicity and lower computational load. As already mentioned, when various reference signatures are used, the ones closer to the operating temperature should be used to get more precise results. Therefore, if there are reference baselines close to the operating temperature, the results may be satisfactory even for nonlinear dependences.

## 8. Conclusions

The present work has two important contributions related to EMI-based SHM systems, regarding the use of steel pipes. First, by modeling using finite elements, the EMI behavior under the temperature effect in two different situations are characterized, changing the values of PZT or/and pipe parameters as a function of temperature. Second, it proposes a new method to compensate the effect of temperature variation. 

Initially, a numerical model was developed using the PZFlex^®^ software. The results obtained from the experimental measurements and from the software were strongly correlated. Therefore, using a simulator, the alterations to the EMI signatures due to temperature variation were investigated and characterized. It was verified that by only changing the parameters of the steel pipe as a function of temperature, there is a frequency shift of the impedance to the left, as the temperature increases, and there is practically no variation in amplitude. However, changing the PZT parameters, it was observed that there are alterations in the EMI signature amplitudes and shifts in frequency. Finally, when changing the parameters of both pipe and PZT, the effects are added to each other. 

Secondly, a new approach was developed to compensate the temperature effect in SHM, with application to steel pipes and by using two computational algorithms. The first one was used to compensate the frequency shifts, which vary linearly depending on the frequency and temperature. The second one was used to compensate the amplitude shifts, which vary linearly according to the temperature. Both algorithms can be easily implemented in any SHM system, embedded or not. Lastly, the metric indices CCDM and RMSD were used for evaluating the damage detection on pipe structures. The method was successfully tested for compensating the temperature effect of two steel pipes, both real and simulated, for temperatures ranging from −40 °C to +80 °C and for a frequency range from 5 kHz to 120 kHz. 

In conclusion, this work conducted a complete analytical, numerical, and experimental study on temperature variation in EMI-based SHM systems applied to pipelines. The study also presented an original and efficient approach based on linear interpolation to compensate the effect of temperature variations in practical applications. The proposed method presents low computational complexity and aims to use a minimum number of previously stored baselines. Even though the tests were performed in steel pipes, the proposed method can be used in any SHM system (EMI-based), provided that the structure presents impedance amplitude and frequency shifts with linear dependence, regardless the structure type. The results obtained for the pipe structures attested the feasibility of the proposed computational algorithm in increasing the precision of damage detection on structures under temperature-varying condition.

Despite the promising results, the need for future works remains to better monitor the structures under the temperature-varying conditions. In addition, the proposed compensation algorithm should be improved to address more effectively the temperature effect compensation in structures that present vertical and horizontal shifts with nonlinear temperature and frequency dependences. Therefore, to achieve this goal, the authors are working on a temperature compensation proposal that uses polynomial interpolation by means of simple equations and low computational load.

## Figures and Tables

**Table 1:** Types of materials and dimensions of the structures, PZTs, and damage.

Material	Name	Dimensions
Steel pipe	pipe-1	Length: 40 cm; external diameter: 4.7 cm; thickness: 1 mm
Steel pipe	pipe-2	Length: 15 cm; external diameter: 12 cm; thickness: 2 mm
PZT patch	PZT-1	Piezo Systems PSI-5A4E model, of 3 cm × 5 mm × 0.508 mm
PZT patch	PZT-2	Piezo Systems PSI-5A4E model, of 3 cm × 7 mm × 0.508 mm
Mass	damage-1	Non-destructive damage: steel mass of 6.5 g, of 12.5 × 11.7 × 6 mm^3^
Mass	damage-2	Non-destructive damage: steel mass of 4 g, of 5 × 5 × 10 mm^3^

**Table 2:** Temperature-dependent parameters used in simulations.

Material	Temperature-Dependent Parameters: Range −40 °C to +80 °C
pipe-1 and pipe-2	Young’s modulus, thermal expansion coefficient, density
PZT-1 and PZT-2	Elastic, piezoelectric, and dielectric coefficients From references [37,38,39]

**Table 3:** Horizontal and vertical shifts of the real part of the impedance between the signatures at −40 °C and +80 °C.

Studied Case	Horizontal Shift of Peaks		Vertical Shift of Peaks	
	18.9–19 kHz	Around 98 kHz	18.9–19 kHz	Around 98 kHz
Case 1	20 Hz	106 Hz	0.39 Ω	2.34 Ω
Case 2	2 Hz	49 Hz	152.25 Ω	115.69 Ω
Case 3	22 Hz	155 Hz	152.35 Ω	118.20 Ω
